# Profiling the Aerobic Window of Horses in Response to Training by Means of a Modified Lactate Minimum Speed Test: Flatten the Curve

**DOI:** 10.3389/fphys.2022.792052

**Published:** 2022-03-22

**Authors:** Lorie De Maré, Berit Boshuizen, Carmen Vidal Moreno de Vega, Constance de Meeûs, Lukas Plancke, Yannick Gansemans, Filip Van Nieuwerburgh, Dieter Deforce, Jean Eduardo de Oliveira, Guilherme Hosotani, Maarten Oosterlinck, Catherine Delesalle

**Affiliations:** ^1^Department of Translational Physiology, Infectiology and Public Health, Research Group of Comparative Physiology, Faculty of Veterinary Medicine, Ghent University, Merelbeke, Belgium; ^2^Equine Hospital Wolvega, Oldeholtpade, Netherlands; ^3^Department of Pharmaceutics, Laboratory of Pharmaceutical Biotechnology, Ghent University, Ghent, Belgium; ^4^Cargill, Research and Development Centre Europe, Vilvoorde, Belgium; ^5^Department of Large Animal Surgery, Anaesthesia and Orthopaedics, Faculty of Veterinary Medicine, Ghent University, Merelbeke, Belgium

**Keywords:** SET, validation, MLSS, fitness, equine, metabolism, lactate, standardised exercise test

## Abstract

There is a great need for objective external training load prescription and performance capacity evaluation in equestrian disciplines. Therefore, reliable standardised exercise tests (SETs) are needed. Classic SETs require maximum intensities with associated risks to deduce training loads from pre-described cut-off values. The lactate minimum speed (LMS) test could be a valuable alternative. Our aim was to compare new performance parameters of a modified LMS-test with those of an incremental SET, to assess the effect of training on LMS-test parameters and curve-shape, and to identify the optimal mathematical approach for LMS-curve parameters. Six untrained standardbred mares (3–4 years) performed a SET and LMS-test at the start and end of the 8-week harness training. The SET-protocol contains 5 increments (4 km/h; 3 min/step). The LMS-test started with a 3-min trot at 36–40 km/h [until blood lactate (BL) > 5 mmol/L] followed by 8 incremental steps (2 km/h; 3 min/step). The maximum lactate steady state estimation (MLSS) entailed >10 km run at the LMS and 110% LMS. The GPS, heartrate (Polar^®^), and blood lactate (BL) were monitored and plotted. Curve-parameters (R core team, 3.6.0) were (SET) VLa_1_._5/2/4_ and (LMS-test) area under the curve (AUC_>/<LMS_), LMS and Aerobic Window (AW) *via* angular vs. threshold method. Statistics for comparison: a paired *t*-test was applied, except for LMS: paired Wilcoxon test; (*p* < 0.05). The Pearson correlation (*r* > 0.80), Bland-Altman method, and ordinary least products (OLP) regression analyses were determined for test-correlation and concordance. Training induced a significant increase in VLa_1_._5/2/4_. The width of the AW increased significantly while the AUC_</>*LMS*_ and LMS decreased post-training (flattening U-curve). The LMS BL steady-state is reached earlier and maintained longer after training. BL_max_ was significantly lower for LMS vs. SET. The 40° angular method is the optimal approach. The correlation between LMS and V_MLSS_ was significantly better compared to the SET. The VLa_4_ is unreliable for equine aerobic capacity assessment. The LMS-test allows more reliable individual performance capacity assessment at lower speed and BL compared to SETs. The LMS-test protocol can be further adapted, especially post-training; however, inducing modest hyperlactatemia prior to the incremental LMS-stages and omitting inclusion of a per-test recovery contributes to its robustness. This LMS-test is a promising tool for the development of tailored training programmes based on the AW, respecting animal welfare.

## Introduction

Because equine competition takes place at an increasing level and frequency, both on a professional and semi-professional level, training horses on a custom-made basis and with maximum efficacy has become of key importance (Serrano et al., 2002). The added value of robust standardised exercise tests (SETs) that show high reproducibility and from which an optimal training load can be derived based upon individual internal load capacity is increasingly recognised in human sports medicine and other animal models, such as dogs and mice. However, most equine athletes are still trained on an empiric basis and a one-approach-fits-for all philosophy (Cunha et al., 2009; Ferasin and Marcora, 2009; Manzo et al., 2009; Alves et al., 2012; Rodrigues et al., 2016; Impellizzeri et al., 2019; Restan and Cerqueira, 2019). Creation of an optimal cardiovascular training programme for each horse without under-/or overtraining is not evident; however, it is of crucial importance, to avoid occurrence of sports injuries and to achieve maximum performance levels (Dyson, 2000; Singer et al., 2008).

With the coming of a wide range of wearables for measuring the external load (speed, distance, etc.) and internal load (heart rate, blood/plasma lactate concentrations, heart rate variability, body temperature, gait symmetry, etc.), the first steps are made towards the objective assessment of performance capacity and individualisation of training programmes (Poole and Erickson, 2008; Peyré-Tartaruga and Coertjens, 2018; Impellizzeri et al., 2019). However, translating the obtained data and the derived performance parameters into effective training advice to induce the desired psychophysiological responses, is rarely applied in the different equestrian disciplines and still needs a lot of optimisation (Bourgela and Blais, 1991; Hauser et al., 2014; Arratibel-Imaz et al., 2016). For this purpose, reliable and reproducible SETs are needed under natural conditions that are easy to implement.

Standardised exercise tests (SETs) can be performed either on a treadmill under laboratory conditions or in the field. The field tests have gained popularity because they better represent the real training and competition conditions and do not cause changes in the locomotion pattern of the horse, a problem that is often encountered during treadmill exercise, especially with unexperienced horses (Courouce, 1999; Allen et al., 2016). Obviously, field tests are more subjected to variance, due to factors such as effect of driver, weather, and track conditions, etc., which entails that test results cannot be extrapolated one on one from the laboratory to the field and vice versa (Courouce, 1999).

The golden standard to assess the aerobic capacity of both human athletes and horses is the Maximum Lactate Steady State (MLSS) (Urhausen et al., 1993). In humans, the MLSS value is defined as the highest constant workload/velocity (V_MLSS_) that can be maintained for at least 25–40 min before anaerobic glycolysis participation starts to increase (Heck et al., 1985; Swensen et al., 1999). This represents the blood lactate steady state condition in between lactate production, removal, and metabolism (Heck et al., 1985; Brooks, 2020). A modified MLSS-test has been designed for horses and is defined as the maximal effort intensity that can be maintained for at least 25–30 min and that induces a maximal blood lactate increase of 1 mmol/L (Gondim et al., 2007; Miranda et al., 2014). Both tests have been shown to be a good predictor for endurance capacity and the level of aerobic fitness and can function as an accurate tool to modulate training loads (Kohrt and O’Connor, 1987; Gondim et al., 2007). However, the downside is that the classical MLSS determination method is not easy to implement in the training schedule of athletes because of the need to perform 4–6 separate exercise sessions at a constant speed, for at least 30 min/session, on consecutive days, to determine the MLSS value (Svedahl and MacIntosh, 2003). Therefore, alternative MLSS protocols have been tested in an attempt to determine the lactate threshold in a different way (shorter 1-day-protocols), likewise alternative SET protocols have been developed in an attempt to approach the MLSS as much as possible in human (Palmer et al., 1999; MacIntosh and Shane, 2002; Lillo-Bevia et al., 2018) and in animal models (Cunha et al., 2009; Rodrigues et al., 2016).

**FIGURE 1:**
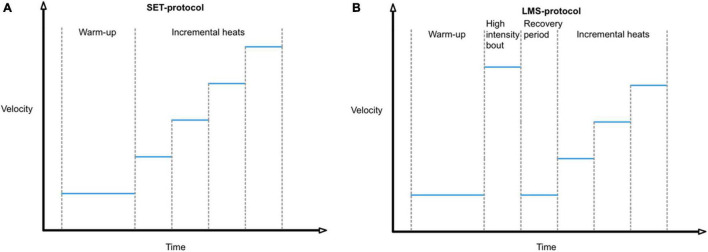
**(A)** Time profile of a typical incremental SET protocol **(B)** vs. LMS (Lactate Minimum Speed) protocol.

In the typical incremental SET protocol that is most often used, horses are, after a warming-up session, subjected to an incremental SET, consisting of consecutive heats of equal duration and increasing speed (Figure 1A; Evans et al., 1993; Davie and Evans, 2000). However multiple difficulties are still encountered with the use of SETs and therefore alternative tests should be investigated.

It is still unclear what the ideal duration of a heat is, which varies between 3 min (Hamlin et al., 2002; Leleu et al., 2005; Gramkow and Evans, 2006) to 5 min (Lindner, 2010b). It is important to maintain each step long enough, so the internal load is assessed in a steady state condition and is representative for that particular speed (De Mare et al., 2017). However, it has been shown that differences in the duration of heats and the amount of steps/test will affect the derived performance parameters in that particular SET protocol (Heck et al., 1985; Foxdal et al., 1996). One recent study in horses showed that the VLa_4_ obtained with SET protocols with shorter heats is generally higher than V_*MLSS*,_ leading to a possible overestimation of aerobic performance capacity (Soares et al., 2014). Besides that, contradicting results can be found in scientific literature with respect to reproducibility, validation, and suitability of these tests to assess aerobic performance capacity for the different equestrian and human sports disciplines. Some studies report a good reproducibility (Dubreucq et al., 1995), whereas others report a lack of scientific evidence (Bourgela and Blais, 1991; Hauser et al., 2014; Arratibel-Imaz et al., 2016). A third and other important factor is the set of parameters that is chosen to indicate the lactate threshold. Many equine SETs use the fixed threshold “VLa_4_,” the velocity at which a blood lactate concentration of 4 mmol/L is measured (Svedahl and MacIntosh, 2003; Wahl et al., 2018). The rationale for using this value lies in a series of studies in which it was shown that with this value, there is a clear correlation between blood and muscle lactate content (Jacobs and Kaiser, 1982). However, this VLa_4_ value that is obtained with such a single session incremental SET is often much higher than the constant sustained speed at which a blood lactate value of 4 mmol/L would have been reached (Svedahl and MacIntosh, 2003). The rigid cut-off value of 4 mmol/L also ignores the pronounced inter-individual differences that exist in production, redistribution, and wash-out capacity for lactate (Svedahl and MacIntosh, 2003). In human athletes, the MLSS ranges from 3–9 mmol/L among individual athletes (Foxdal et al., 1996; MacIntosh and Shane, 2002). Likewise, in horses, there is increasing evidence that a fixed cut-off value for blood lactate is not a reliable predictor for the lactate threshold (Gondim et al., 2007; Miranda et al., 2014). It is thus important to realise that using VLa_4_, as threshold parameter in incremental SETs, can lead to the overestimation of MLSS and thus, to the implementation of unrealistic high training velocities and intensities with all its negative consequences (Beneke, 1995; Gondim et al., 2007; Soares et al., 2014). Indeed, Lindner (2010a) demonstrated that VLa_2_ could not be maintained for 30 min without blood lactate increases of >1 mmol/L, and the MLSS of horses corresponds more to a VLa_1_._5–2_. Therefore, VLa_4_ is not deemed to be a suitable parameter to predict the MLSS in horses (Gondim et al., 2007; Lindner, 2010a). With that respect, controversy has been reported to exist regarding the correlation between VLa_2_ or VLa_4_ and V_MLSS_ (Heck et al., 1985; Aunola and Rusko, 1992).

Besides these fixed cut-off values, another way to approach the obtained SET curve in order to derive SET parameters is the D_max_ and Modified D_max_ method.

For the D_max_ method, exercise tests are performed until exhaustion, which is difficult in field tests. A third order curvilinear regression curve is constructed based on blood/plasma lactate concentrations vs. workload (Zhou and Weston, 1997). A line is drawn in between the starting and end point of the lactate curve. The inflexion point at which the distance between the line and the lactate regression curve is maximal is annotated as the D_max_ value, and represents the threshold point at which a steady-state is reached between lactate production and elimination (Zhou and Weston, 1997). Human studies report that the D_max_ approach is suitable to define the individual lactate threshold to exercise and provides a good estimation of the MLSS (Zhou and Weston, 1997; Siahkouhian et al., 2012; Zwingmann et al., 2019). In contrast, a study of Janeba et al. (2010), concluded that the D_max_ approach is not a valid method because of the high dependence on the lowest and highest workload data points of the lactate curve (Janeba et al., 2010). Therefore, the Modified D_max_ has been developed, though controversies regarding its ability or inability to estimate the MLSS remains (Janeba et al., 2010; Siahkouhian et al., 2012; Płoszczyca et al., 2020).

Therefore, besides the SET protocol, the mathematical approach that is used to derive performance parameters and training loads from the lactate-velocity curve is important. Multiple human studies try to define zones with upper and lower limits (aerobic, anaerobic, and a transitional zone) by assessing and combining internal load parameters (heart rate, ventilatory, and lactate parameters) with the external load (Wyon and Redding, 2003; Santos et al., 2010; Clemente Suárez and González-Ravé, 2014; von Haaren et al., 2015; Rogers et al., 2021).

**FIGURE 2:**
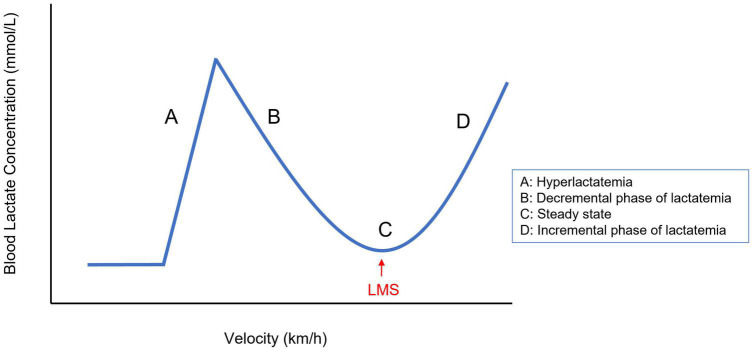
Typical shape of the LMS curve illustrating the consecutive phases **(A–D)** of the LMS protocol. The LMS is marked by an arrowhead. LMS, lactate minimum speed.

A SET type that could be a good alternative and that has been studied in only a few equine trials is the lactate minimum speed test (LMS test), which is the only single session test that is based on challenging the lactate steady state of an individual athlete, similar to the MLSS test (Wahl et al., 2018). The LMS test has originally been developed for human athletes by Tegtbur et al. (1993) to estimate the Anaerobic Threshold (AT) and MLSS at the same time (Tegtbur et al., 1993). This test consists of 3 consecutive phases: a short exercise bout of high intensity to induce hyperlactatemia, followed by a passive recovery period of approximately 8 min to provide time for lactate transposition from cells to the bloodstream to create a steady peak-blood lactate concentration, followed by the third and last phase, an incremental exercise test (Figure 1B). The LMS test produces a typical *U*-shaped blood lactate time-profile (Figure 2). In the first stages of the decremental section of the blood lactate vs. speed curve, there is a net blood lactate clearance with a decrease in blood lactate concentration until lactate production exceeds its removal. The lowest point of this *U*-shaped curve corresponds with the velocity at which the blood lactate concentration is minimal and represents the point of balance between lactate clearance and production. In human sports medicine this test is reported to reliably indicate the lactate threshold for different sports disciplines such as running, rowing, cycling, and swimming (MacIntosh and Shane, 2002; Ribeiro et al., 2003; Sotero et al., 2009; Knoepfli-Lenzin, 2011; Campos et al., 2014). The test enables the estimation of the MLSS based on only one short exercise session which makes this test more practically applicable than others (Tegtbur et al., 1993; Bacon, 1999).

A study by Gondim et al. (2007) validated this LMS test in five endurance horses, which were subjected to a 500 m full gallop exercise on a grass track, followed by a recovery period of variable duration, and subsequent incremental exercise bouts of 1,000 m, starting at 15 km/h with increments of 2 km/h per heat (Gondim et al., 2007). The data were plotted and the point at which lactate concentration showed an inflexion was annotated as the LMS value, which corresponds with the lowest point of the polynomial fitted U-curve (see Figure 2). After LMS determination, horses were subjected to two 10,000 m runs, one at LMS and one at 10% above LMS to test LMS accuracy. The applied LMS protocol was reported to be reliable to estimate the MLSS in endurance horses (Gondim et al., 2007).

A second study performed by Miranda et al. (2014) compared 5 different LMS protocols executed on a treadmill with 8 purebred Arabian horses to evaluate protocol-dependency of obtained test results, since human studies clearly indicate protocol dependency (Carter et al., 1999; Ribeiro et al., 2009). Each of the 5 protocols consisted of 6 incremental steps with varying incremental speeds (ranging from 1.8–3.6 km/h) and stage length (ranging from 3–7 min/step). The study showed a high correlation (*r* = 0.7) between MLSS velocities and the obtained LMS values when using the protocol with incremental bouts of 3 min, starting at 14.4 km/h with 1.8 km/h increments (Miranda et al., 2014).

Finally, Soares et al. (2014), tested a LMS protocol on a 5% inclined treadmill and compared this with the VLa_2/4_ and the MLSS (Soares et al., 2014). They found a good correlation between the MLSS velocity and respectively the VLa_2/4_ (*r* = 0.74/0.78) and LMS (0.83), however the VLa_2_ and VLa_4_ were on all occasions much higher compared to the MLSS-velocity and horses were not able to maintain this speed for a long time (Soares et al., 2014). The concordance between protocols was relatively poor.

The LMS-test is a promising exercise test to assess the individual blood lactate steady state without performance until exhaustion; however, currently, only very few studies are available in horses focusing on LMS testing (Gondim et al., 2007; Miranda et al., 2014; Soares et al., 2014). Only one in the field study is available and that study includes a recovery period after the initial hyperlactatemia inducing exercise phase into the applied LMS protocol (Gondim et al., 2007). No studies are available looking into the most optimal mathematical approach to deduce performance parameters from the obtained LMS test curves. No studies are available comparing SET vs. LMS deduced performance parameters in the field and no study has been looking into the effect of training on evolution of LMS test performance parameters. Lastly, almost no studies combine these internal and external loads in order to obtain objective training loads that create the most optimal psychophysical response (Impellizzeri et al., 2019).

Therefore, the aim of the current study was threefold: (1) to compare the performance parameters of a classic incremental SET with multiple new performance parameters deduced from a modified LMS-test protocol; (2) to assess the effect of training on LMS test parameters and curve shape and to compare this with the SET parameters; (3) and to identify the most optimal mathematical approach for LMS curve parameter assessment to deduce objective and optimal training loads. To our knowledge, this is the first equine study assessing not only the effect of training on the LMS value but also, the individual aerobic window of each horse.

## Materials and Methods

Six untrained standardbred mares, aged between 3 and 5 years were trained 4 days/week for 8 consecutive weeks. The horses were stabled at the same training facility and trained on the same racetrack by the same dedicated person. Horses were comparable in body condition score (4–5/9) and body weight (418–490 kg). All mares underwent a pre-purchase examination (clinical examination and radiographies) to detect or rule out any significant pathology in advance. Objective proof of the absence of lameness was determined by the Equinosis Q system analyses with the Lameness Locator Software (Lameness Locator 2014 v.2, Equinosis^®^) for all horses before the start and at the end of the training period (Broeckx et al., 2019). Horses were housed in individual boxes on straw bedding and received concentrate feed two times daily and were provided with *ad libitum* access to tap water and roughage. There was an acclimation period of 2 weeks before the start of the training trial. Throughout the entire trial, horses were monitored two times daily for heart rate, respiratory rate, capillary refill time, appetite, rectal temperature, and faecal output and consistency by a dedicated veterinarian. This study was approved by the Animal Ethics Committee of the Ghent University (EC 2016/40) and was partly funded by VLAIO AIOONO20160007.

### Exercise Protocols

The horses performed, at random, an LMS test or SET on 2 separate days on average, 1 week in between, before the start of the training programme and after the training period of 8 weeks. All mares performed the same LMS vs. SET protocol. All tests were undertaken while the horse was harnessed ridden by the same trainer on the same oval-shaped sand racetrack. Horses were equipped with an HR-monitor (Polar^®^ Equine H7, Polar Electro Oy, Finland) to assess heart rate and speed. Blood lactate concentration was measured at rest and at 1 min-walking intervals in between the exercise bouts. Blood samples were taken from the left jugular vein and whole blood lactate concentration was immediately determined by a hand-held lactate analyser (Lactate Pro 2, Axon Lab AG^®^) (Arratibel-Lmaz et al., 2017; Crotty et al., 2021).

#### Standardised Exercise Test Protocol

The SET consisted of a 10-min warm-up at 20 km/h followed by 5 incremental exercise bouts of 3 min duration, with a 1-min walk interval in between to allow for blood sampling. An increase of 4 km/h for every exercise step was applied until a velocity of 40 km/h was reached. After the end, blood samples were taken every 15 min at rest until blood lactate concentration returned to pre-exercise levels. The track was watered and raked on each occasion to maximally assure equal study conditions throughout the trial.

##### Standardised Exercise Tests Parameters

The relationship between velocity and lactate was plotted by means of an exponential regression curve to determine following SET parameters: the VLa_1_._5/2/4_ (velocity at a blood lactate concentration of, respectively, 1.5/2/4 mmol/L).

#### Lactate Minimum Speed Test Protocol

The LMS-test protocol consisted of a 10-min warm-up at 20 km/h followed by a fast 3-min trot at 36–40 km/h to induce hyperlactatemia (a blood lactate >5 mmol/L), followed by 8 incremental steps of 3 min, increasing speed with 2 km/h/step starting from 20 to 34 km/h. Blood was sampled after each step.

##### Mathematical Lactate Minimum Speed Curve Approach to Determine Lactate Minimum Speed Parameters

**FIGURE 3:**
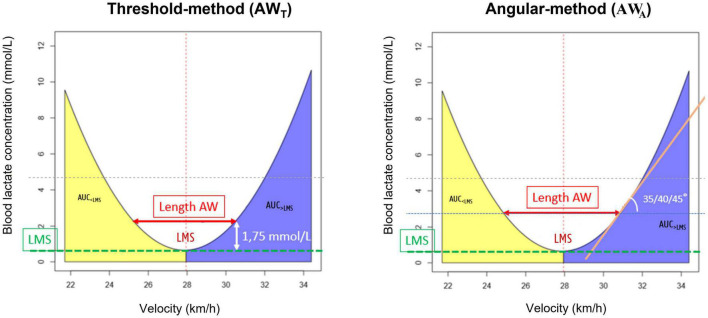
Mathematical approaches to determine the aerobic window from the LMS-test. Left Panel: aerobic window calculation, using the threshold method; Right Panel: aerobic window calculation, using the angular method. AW, aerobic window; LMS, lactate minimum speed; AUC_<LMS_, area under the curve at the left side of the LMS; AUC_>LMS_, area under the curve at the right side of the LMS.

To determine different individual LMS-test parameters, a second-degree polynomial function curve fitting was performed on the blood lactate concentration vs. speed values using the R software (v3.6.0, R core team, 2019). The following curve-parameters were determined (Figure 3): LMS, Area under the curve on the left side of the LMS (AUC_<LMS_ ∼ Lactate wash-out) and right side of the LMS (AUC_>LMS_ ∼ Lactate accumulation) and magnitude of the aerobic window (AW). The lowest point of the polynomial *U*-shaped curve corresponds to the LMS value, which was calculated using the equation of the fitted polynomial.

The aerobic window (AW) for each LMS test, was assessed by means of 2 different mathematical approaches (threshold and angular method) and compared with the individual SET derived lactate threshold of each horse. Calculations were done in Microsoft Excel using the equation of the fitted curve. The AW represents the speed range within which blood lactate concentrations remain stable and corresponds with the interval between the upper and lower limit on respectively the right and left side of the LMS value. Two different mathematical approaches were applied to determine these limits: For the threshold method (AW_T_), the limits were set at speeds that corresponded with LMS plus and minus speeds at blood lactate concentration of respectively 1/1.25/1.5/1.75/2 mmol/L (Figure 3). For the angular method (AW_a_), the limits corresponded with the speeds indicated at the intersection point between the horizontal line through the LMS and the tangent at an angle of respectively 35/40/45 degrees (Figure 3). These mathematical methods were investigated on both the individual level as well as across all horses.

#### Validation of Lactate Minimum Speed

Validation of the obtained LMS value as estimation of the MLSS was performed as described by Gondim et al. (2007). Briefly, horses were subjected to two different exercise protocols on two separate days, after a warm-up for 10 min at 20 km/h, a first exercise session of ≥10 km at a constant LMS speed (day 1) and a second exercise session at 110% of LMS (day 2) was performed. Blood lactate concentration was measured every 2 km. If the venous blood lactate concentration increased >1 mmol/L at 110% LMS however not at LMS itself, than 100% LMS was considered as a good estimation of the V_MLSS_. If the horses increased >1 mmol/L in blood lactate concentration at 100% LMS or if horses did not increase with >1 mmol/L at 110% LMS, then a third run was introduced.

### Training

Each training session consisted of a warming-up period of 10 min jogging (±20 km/h) followed by either aerobic or interval training. The speed for the training sessions was based on the horses’ individual LMS. The mares performed 2–3 days/week 30 min of aerobic training (±22–25 km/h ∼ 90%LMS, with heart rate values (HR) between 150 and 160 bpm using the Polar^®^ watch during the training as guidance) and 1–2 days/week interval training (three times 3 min at high speed in between 30 and 35 km/h ∼ 130%LMS, with HR values between 180 and 200 bpm as guidance). In between the high-speed intervals, the mares were trotted each time for 3 min at aerobic speed (90%LMS).

### Data Processing and Statistical Analysis

Each performance parameter, for SET and LMS, was compared before vs. after 8 weeks of training. Statistical analysis was conducted in R (v3.6.0, R core team, 2019). Normality of the data was determined using a Shapiro-Wilk test. A paired samples *t*-test was applied for normally distributed data, otherwise, a paired samples Wilcoxon test was applied. Of all data, the means and standard deviations are presented with extra individual data of the aerobic window. Correlations between the tests were determined using the Pearson correlation coefficient. Statistical significance was set at *p* < 0.05 and *r* > 0.80. The relationship between the velocities predicted by the SET (VLa_1_,_5/2/4_) or LMS-test was compared to the V_MLSS_ using the Bland-Altman method as well as ordinary least products regression (OLP). For the Bland-Altman method, the mean bias and the limits of agreement (mean ±1.96 SD) were calculated (Ludbrook, 2002). Agreement was predefined as an absolute mean bias of 1 km/h and limits of agreement (95% CI) within 1.5 km/h of the mean difference. OLP was used to determine the fixed and proportional bias between two variables. A fixed bias was present when the 95% CI for the y-intercept included 0 in this interval. A proportional bias was present if the 95% CI for the regression slope included 1 in this interval (Ludbrook, 2002).

## Results

No adverse events (daily clinical monitoring) occurred during this 8-week training period. All mares completed the training trial in good health. No signs of lameness were detected visually nor by means of the Equinosis Q System with Lameness Locator Software (Equinosis^®^) throughout the entire training trial (Lameness Locator 2014 v.2, Equinosis^®^). All mares performed the same prescribed exercise protocol. With respect to the number of performed heats: one mare did not perform the 32 and 34 km/h LMS-incremental-step before training and 3 mares did not perform the 34 km/h LMS-step after training. Heart rate values obtained during aerobic training were: HR_mean_ 153.3 ± 12.8 bpm, HR_min_ 119.4 ± 17.4 bpm, HR_max_ 180.1 ± 26.8 bpm. HR values during interval training consisting of 3 heats at high speed were the following: Heat 1: HR_mean_ 169 ± 35.5 bpm, HR_min_ 153.5 ± 42.2 bpm, HR_max_ 188.3 ± 24.4 bpm/Heat 2: HR_mean_ 177.0 ± 45.2 bpm, HR_min_ 156.5 ± 43.8 bpm, HR_max_ 185.3 ± 29.7 bpm/Heat 3: HR_mean_ 185.4 ± 27.7 bpm, HR_min_ 155.4 ± 38.2 bpm, HR_max_ 210.6 ± 8.5 bpm.

### Standardised Exercise Tests-Curve Parameters

**FIGURE 4:**
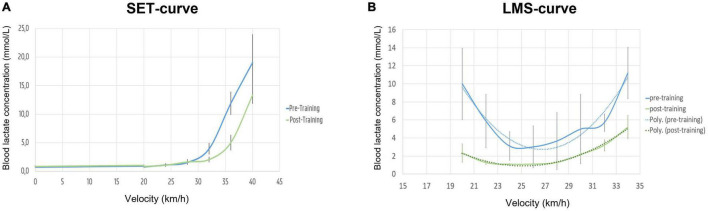
Evolution of velocity vs. blood lactate concentration pre- and post-training (*n* = 6 horses). **(A)** Standardised exercise test curves; **(B)** lactate minimum speed curves. Poly-, polynomial curve fitting.

A shift to the right of the exponential curve was seen for all mares after 8 weeks of training (see Figure 4A and Table 1) with a significant increase in VLa_1_._5/2/4_ (*p* = 0.007/0.007/0.012).

**TABLE 1:** Overview of obtained SET vs. LMS parameters before and after 8 weeks of training.

	SET-curve parameters			LMS-curve parameters	
	Before training	After training		Before training	After training
VLa_1_._5_ (km/h)	26.8 ± 1.8	29.7 ± 1.2	AUC_<LMS_	1.9 ± 0.9	0.5 ± 0.2
VLa_2_ (km/h)	28.3 ± 1.6	31.5 ± 1.2	AUC_>LMS_	2.9 ± 0.8	1.2 ± 0.3
VLa_4_ (km/h)	31.5 ± 1.2	34.3 ± 1.4	LMS (km/h)	26.7 ± 1.0	25.4 ± 0.9

### Lactate Minimum Speed-Curve Parameters

The parabolic LMS-curve flattens after 8 weeks of training (Figure 4B) due to a significant decrease in AUC_<LMS_ (*p* = 0.020) and AUC_>*LMS*_ (*p* = 0.008) (Table 1) combined with a significant increase in the width of the AW (Tables 2–4) and a significant decrease of the LMS after 8 weeks of training (*p* = 0.008).

**TABLE 2:** Assessment of the width of the aerobic window of each horse (UL minus LL), using the angular approach (AW_a_) at tangent angles of respectively 35, 40, and 45°.

			Horse 1	Horse 2	Horse 3	Horse 4	Horse 5	Horse 6
Lactate at LMS	Before	(mmol/L)	1.1	1.4	6.3	1.7	1.0	1.9
	After	(mmol/L)	0.8	0.7	1.0	0.6	0.9	1.4
LMS	Before	(km/h)	27.1	27.6	26.0	24.8	27.7	27.0
	After	(km/h)	25.5	26.2	23.6	24.8	26.4	25.6
AW_a–35^°_	Before	Width AW	3.2	2.9	3.9	8.3	3.9	4.5
		Velocity at UL (km/h)	28.6	29.1	27.9	29.1	29.6	29.3
		Velocity at LL (km/h)	25.5	26.2	24.0	20.7	25.6	24.8
		Lactate at UL (mmol/L)	1.7	1.9	7	3.2	1.7	2.7
	After	Width AW	12.5	10.2	21.1	11.1	13.1	8.4
		Velocity at UL (km/h)	31.8	31.3	34.3	30.4	33.0	29.9
		Velocity at LL (km/h)	19.3	21.1	13.2	19.3	19.9	21.5
		Lactate at UL (mmol/L)	3.0	2.5	4.7	2.5	3.2	2.9
AW_a–40^°_	Before	Width AW	3.8	3.5	4.7	10.0	4.72	5.4
		Velocity at UL (km/h)	29.0	29.3	28.3	29.9	30.0	29.8
		Velocity at LL (km/h)	25.2	25.9	23.6	19.9	25.2	24.4
		Lactate at UL (mmol/L)	1.9	2.2	7.3	3.8	2.0	3.1
	After	Width AW	15.0	12.2	25.3	13.3	15.7	10.1
		Velocity at UL (km/h)	33.1	32.3	36.4	31.5	34.3	30.8
		Velocity at LL (km/h)	18.1	20.1	11.1	18.2	18.6	20.7
		Lactate at UL (mmol/L)	3.9	3.3	6.3	3.4	4.2	3.5
AW_a–45^°_	Before	Width AW	4.5	4.1	5.6	11.9	5.6	6.4
		Velocity at UL (km/h)	29.3	29.7	28.8	30.9	30.4	30.3
		Velocity at LL (km/h)	24.8	25.5	23.1	19.0	24.8	23.8
		Lactate at UL (mmol/L)	2.2	2.5	7.7	4.7	2.4	3.5
	After	Width AW	17.9	14.6	30.1	15.8	18.7	12.0
		Velocity at UL (km/h)	34.5	33.5	38.8	32.7	35.8	31.7
		Velocity at LL (km/h)	16.6	18.9	8.7	16.9	17.0	19.7
		Lactate at UL (mmol/L)	5.3	4.3	8.6	4.5	5.6	4.4

*UL, upper limit; LL, lower limit.*

**TABLE 3:** Assessment of the aerobic window across all horses using both mathematical approaches: the threshold (AW_T_) and the angular (AW_a_) approach.

	AW_T_					AW_a_		
	AW_T–1_	AW_T–1.25_	AW_T–1.5_	AW_T–1.75_	AW_T–2_	AW_a–35^°_	AW_a–40^°_	AW_a–45^°_
Width AW_<8w_	4.8 ± 1.1	5.4 ± 1.1	6.0 ± 1.2	6.4 ± 1.2	6.7 ± 1.5	4.4 ± 1.7	5.3 ± 2.0	6.3 ± 2.4
Width AW_>8w_	8.4 ± 1.3	9.4 ± 1.4	10.3 ± 1.6	11.2 ± 1.7	11.9 ± 1.8	12.7 ± 4.0	15.3 ± 4.8	18.1 ± 5.8
*P*-value < 8w vs. >8w of training	0.005	0.005	0.004	0.008	0.004	0.01	0.01	0.01

**TABLE 4:** The mean bias and the 95% limits of agreement for the Bland-Altman method and the Pearson’s correlation coefficient (*r*- and *p*-value) between the V_MLSS_ and the LMS/VLa_1_._5_,_2_,_4_ before and after 8 weeks of training.

	Before 8 weeks of training				After 8 weeks of training			
	Mean bias (km/h)	95% limits of agreement (km/h)	*r*-value	*p*-value	Mean bias (km/h)	95% limits of agreement (km/h)	*r*-value	*p*-value
V_MLSS_-LMS	1.04	−0.40 to 2.47	0.93	0.007	−0.97	−2.57 to 0.63	0.65	0.16
V_MLSS_-VLa_1_,_5_	1.12	−3.83 to 6.06	0.02	0.97	2.48	−0.64 to 5.6	−0.75	0.07
V_MLSS_-VLa_2_	2.48	−0.64 to 5.60	−0.0045	0.99	4.14	1.58 to 6.70	−0.33	0.53
V_MLSS_-VLa_4_	5.86	1.77 to 9.95	−0.0070	0.99	7.11	5.38 to 8.84	0.59	0.22

The maximum blood lactate concentration (BL_max_) was significantly lower during the LMS-test (mean BL_max < 8w training_ was 12.9 ± 3.1 mmol/L and BL_max > 8w training_ was 5.4 ± 1.0 mmol/L) compared to the conventional SET (mean BL_max < 8w training_ was 19.1 ± 4.9 mmol/L and BL_max>8w training_ was 14.5 ± 1.4 mmol/L) (*p* = 0.002 before training/*p* < 0.0002 after training). The maximum blood lactate concentration was lower for both tests after training vs. before. The mean blood lactate concentration at LMS was significantly lower after 8 weeks of training (*p* = 0.031) with a mean of 2.2 ± 1.8 mmol/L before and 0.9 ± 0.3 mmol/L after training.

A significant increase after training in the width of the AW was seen for both the threshold and angular mathematical approach method for each horse (see Tables 2, 5) and across all horses (see Table 3). This was more pronounced for the angular method compared to the threshold method (see Table 3).

**TABLE 5:** Assessment of the width of the aerobic window (UL minus the LL) of each horse, by means of the threshold approach (AW_T_) at blood lactate concentration of respectively 1/1.25/1.5/1.75 and 2 mmol/L.

			Horse 1	Horse 2	Horse 3	Horse 4	Horse 5	Horse 6
LMS	Before	(km/h)	27.1	27.6	26.0	24.8	27.7	27.0
	After	(km/h)	25.5	26.2	23.6	24.8	26.4	25.6
AW_T–1_	Before	Width AW	4.3	4.1	3.6	6.9	4.7	5.1
		Velocity at UL (km/h)	29.2	29.6	28.4	28.4	30.0	29.6
		Velocity at LL (km/h)	24.9	25.6	24.8	21.5	25.2	24.5
	After	Width AW	8.3	7.6	10.98	7.9	8.7	6.9
		Velocity at UL (km/h)	29.8	30.0	29.2	28.8	30.8	29.2
		Velocity at LL (km/h)	21.6	22.4	18.2	20.9	22.1	22.2
AW_T–1.25_	Before	Width AW	4.8	4.5	4.3	7.7	5.3	5.7
		Velocity at UL (km/h)	29.4	29.9	28.7	28.8	30.2	29.9
		Velocity at LL (km/h)	24.7	25.3	24.5	21.1	24.9	24.2
	After	Width AW	8.5	8.5	12.3	8.8	9.7	7.8
		Velocity at UL (km/h)	30.3	30.4	29.9	29.2	31.3	29.6
		Velocity at LL (km/h)	20.9	21.9	17.6	20.4	21.6	21.8
AW_T–1.5_	Before	Width AW	5.4	5.0	5.0	8.5	5.8	6.2
		Velocity at UL (km/h)	29.7	30.1	29.0	29.1	30.5	30.2
		Velocity at LL (km/h)	24.3	25.1	24.0	20.7	24.7	23.9
	After	Width AW	10.4	9.3	13.4	9.7	10.6	8.5
		Velocity at UL (km/h)	30.8	30.9	30.5	29.7	31.7	30.0
		Velocity at LL (km/h)	20.4	21.5	17.0	20.0	21.2	21.5
AW_T–1.75_	Before	Width AW	5.6	5.4	5.25	9.1	6.3	6.7
		Velocity at UL (km/h)	29.9	30.3	29.2	29.5	30.7	30.4
		Velocity at LL (km/h)	24.3	24.9	23.9	20.3	24.4	23.7
	After	Width AW	11.2	10.1	14.5	10.5	11.5	9.2
		Velocity at UL (km/h)	31.2	31.2	31.0	30.1	32.2	30.3
		Velocity at LL (km/h)	20.0	21.1	16.5	19.5	20.7	21.1
AW_T–2_	Before	Width AW	6.0	5.7	5.7	9.8	5.7	7.2
		Velocity at UL (km/h)	30.1	30.5	29.4	29.8	31	30.6
		Velocity at LL (km/h)	24.1	24.7	23.7	20.0	24.2	23.5
	After	Width AW	12.0	10.8	15.5	11.3	12.2	9.8
		Velocity at UL (km/h)	31.6	31.6	31.5	30.4	32.5	30.6
		Velocity at LL (km/h)	19.6	20.8	16.0	19.2	20.3	20.8

*UL, upper limit; LL, lower limit.*

The width of the AW is comparable before 8 weeks of training for both calculation methods; however after training, the width of the AW is larger on all occasions for the angular method, when compared to the threshold method. The shape of the parabolic curve remained stable for most horses within a range of 2–4 mmol/L above the lactate concentration at LMS however varied among individuals whereas the threshold concept ignores individual differences in the shape of the curve and therefore identifies a smaller AW post training. Within the angular method, the 35° angle approach seems less discriminative because only minor differences in lactate concentration occur between the lactate concentration at the upper limit of the AW vs. blood lactate concentration at the LMS (see Table 2). Likewise, results obtained with the 45° angle approach are not reliable and tend to overestimate the AW of horses. A difference in blood lactate concentration as high as 7.53 mmol/L was observed between the lactate concentration at the upper limit of the AW vs. at LMS, for example, for horse 3 after 8 weeks of training (see Table 2). Comparable results were obtained for the other horses (3–4.7 mmol/L). Therefore, the 40° angle seems to be the most optimal discriminant approach to assess the AW of sport horses without overestimating the width of the AW.

### Lactate Minimum Speed-Validation

**FIGURE 5:**
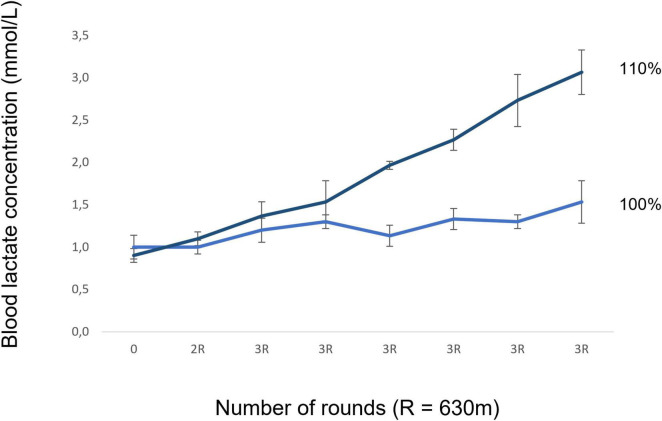
Validation of the LMS test at speeds of respectively 100% LMS and 110% LMS. No blood lactate increase (>1 mmol/L) occurred during validation at 100% LMS; however, it did so at 110% LMS for most of the mares.

No blood lactate increase (>1 mmol/L) occurred during validation at 100% LMS and at 110% LMS blood lactate concentration increased over 1 mmol/L for most of the mares (see Figure 5). Only 3 horses needed one extra run for MLSS determination, whereas one before and two after 8 weeks of training. A very high correlation (see Table 4) was seen between the LMS and the V_MLSS_ especially before the training period (with a mean LMS_<8*w*_ of 26.7 ± 1.0 km/h vs. a mean MLSS_<8*w*_ of 25.7 ± 1.5 km/h). Likewise, a high correlation was seen between LMS and V_MLSS_ after 8 weeks of training (with a mean LMS_>8*w*_ of 25.3 ± 1.8 km/h vs. a mean MLSS_>8*w*_ of 26.5 ± 1.1 km/h). A better correlation was observed between the LMS and V_MLSS_ than between the V_MLSS_ and the VLa_1_._5/2/4_ before and after 8 weeks of training (see Table 4).

**FIGURE 6:**
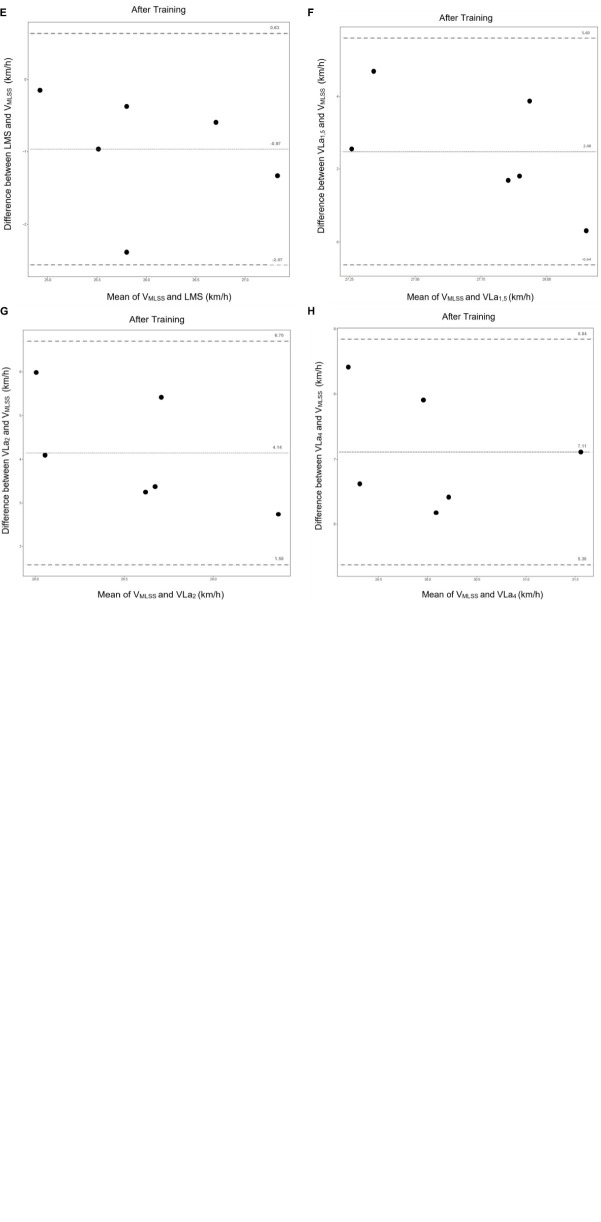
Bland-Altman plots with the mean bias and the 95% agreement CI for comparison between velocities of the MLSS vs. LMS **(A,E)**, VLa_1_,_5_ **(B,F)**, VLa_2_ **(C,G)**, and VLa_4_ **(D,H)** with **(A–D)** before the 8 weeks of training and **(E–H)** after 8 weeks of training. For each plot: the dotted line in the centre represents the mean bias and the upper and lower dotted line delimit of the 95% limits of agreement for the mean bias.

Bland-Altman limits of agreement plots (see Figure 6) show the difference between velocities obtained with the LMS-test or SET compared to the V_MLSS_. The agreement between the velocities is expressed in terms of mean bias and 95% limits of agreement. The best agreement was found between the V_MLSS_ and the LMS, before and after training, followed by the VLa_1_,_5_. Before training, only a mean bias of 1.04 km/h (−0.40 to 2.47 km/h) was seen between the LMS and V_MLSS_, whereas −0.97 km/h (−2.57 to 0.63 km/h) after training. The OLP regression analyses results are shown in Table 6.

**TABLE 6:** Ordinary least products regression results for the V_MLSS_ vs. the LMS/VLa_1_._5_,_2_,_4_ before and after 8 weeks of training.

	2 variables	Y-intercept (95% CI)	Slope (95% CI)	Proportional bias	Fixed bias
Before training	V_MLSS_-LMS	9.46 (2.81–16.10)	0.67 (0.41–0.93)	Yes	Yes
	V_MLSS_-VLa_1_,_5_	−3.98 (−36.32–28.36)	1.20 (−0.06–2.46)	No	No
	V_MLSS_-VLa_2_	56.71 (26.85–86.58)	−1.11 (−2.27–0.05)	Yes	Yes
	V_MLSS_-VLa_4_	51.74 (30.49–73.00)	−0.79 (−1.61–0.04)	Yes	Yes
After training	V_MLSS_-LMS	4.03 (−13.16–21.22)	0.81 (0.16–1.50)	No	No
	V_MLSS_-VLa_1_,_5_	45.29 (33.93–56.65)	−0.62 (−1.04–0.19)	Yes	Yes
	V_MLSS_-VLa_2_	43.45 (30.74–56.15)	−0.48 (−0.96–0.004)	Yes	Yes
	V_MLSS_-VLa_4_	12.01 (−6.38–30.40)	0.82 (0.12–1.50)	No	No

## Discussion

This study tested a modified LMS protocol in field conditions and is the first equine study that compared obtained curve parameters with those obtained with a classical incremental SET protocol in the field. It is the first equine study to assess the effect of training on LMS test parameters and to explore different mathematical approaches to determine LMS test curve parameters. Study results show that the LMS test is a promising method to assess individual performance parameters in horses at lower blood lactate concentrations and external loads when compared to the classical SETs. This is most probably because challenging an individual physiological steady state is a much more realistic approach when compared to the use of pre-set cut off values, as indicators for physiological target points such as the time point at which the shift occurs from predominant aerobic towards anaerobic metabolism.

All horses showed a significant increase in VLa_1_._5/2/4_ after 8 weeks of training based upon classical incremental SET results, which correlates with an increased aerobic performance capacity. The 9% increase in VLa_4_ after 8 weeks of training is in accordance with the 2–35% increase, reported in other studies (Sotero et al., 2009). The increase in speed that can be maintained without increase in blood lactate concentration is something that can be expected when the horses are better trained. As a result of training, horses will rise less quickly in lactate concentration and will therefore be able to reach higher speeds at these fixed lactate values. A study by Bourgela and Blais (1991), showed that the reproducibility of VLa_4_ of incremental SETs in field conditions is not acceptable from a scientific point of view (Bourgela and Blais, 1991). Thus, one might question how reliable and sensitive this value is to monitor training progression of a specific horse? This should also be taken into account when using the VLa_4_ value to create and evaluate a training schedule. As mentioned previously, the use of VLa_4_ in field conditions, predisposes to overestimation of performance capacity of a tested horse, and therefore formulation of training advice at speeds and heart rates that actually are too high for that particular horse. Similar results were obtained in this field study where the Bland-Altman plots showed clearly that the VLa_4_ overestimates the V_MLSS_ and even the VLa_2/1_,_5_ when compared to the LMS-test on each occasion (see Figure 6 and Table 4). The VLa_1_,_5_, however, is the lactate threshold deduced from SETs that appropriates the most the V_MLSS_ before and after training, which confirms similar results obtained in a recent equine study (Lindner, 2010a). The correlation between SET-parameters and the V_MLSS_ was, however, low and often negative (see Table 4). The LMS test, however, pushes the body of the horse into a steady state, which is subsequently challenged. This test deduces performance capacity by challenging many complex biological feedback mechanisms that are involved in performance capacity, at the same time. In other words, it is an approach that takes much more into account the dynamics of many physiological processes. The LMS test also allows for assessment of the width of the aerobic window (speed range where exercise occurs, fuelled by predominant aerobic metabolism) of a specific horse, which obviously is of more value than determination of a single threshold value based upon classical incremental SET results.

Training clearly flattens the *U*-shaped LMS test curve: the LMS blood lactate steady state is reached at an earlier stage and is maintained for a longer period and at higher velocities when compared to pre-training conditions. This is illustrated by a significant decrease of AUC_<LMS_, AUC_>*LMS*_, and LMS value, combined with a significant increase in the width of the AW. Widening of the aerobic window is the illustration of occurrence of adaptive physiological processes amongst which an improved lactate clearance capacity due to complex physiological processes such as upregulation of cellular expression of lactate transporters and improved utilisation of lactate as energy source in the first incremental stages of exercise (Brooks, 2020). The latter results in the unfolding of the *U*-shaped LMS curve and a decrease of the LMS value after training (Donovan and Brooks, 1983). Most probably, the widening of the AW also indicates a shift towards preferential use of alternative metabolites to fuel muscle exercise. However, more research is needed with that respect. By having an exact view on the width of the aerobic window, trainers know exactly within which boundaries a certain horse is predominantly challenged from an aerobic vs. anaerobic point of view. This knowledge can greatly optimise efficacy of training programmes, creating a better match between time dedicated to training, strain imposed on the musculoskeletal system and obtained competition results. More future research about training programmes based on this AW would be interesting.

Study results also showed that the mathematical approach that is chosen to deduce performance parameters is crucial to obtain meaningful information. As expected, the threshold method is least reliable, since it is based upon application of pre-set cut-off values which obviously hinders an individualised approach and takes less into account the individual differences in metabolic kinetics. With respect to the angular approach, the 40° angular approach seems the most reliable physiological approach because the 35° approach is less discriminative, however the 45° approach is too discriminative. The high blood lactate concentrations reached at the UL of the 45° method for several mares, could not represent a mainly aerobic training window across all horses. Overestimation of the speed-range (AW) at which the transition between mainly aerobic and anaerobic exercise occurs, leads to training advise that can lead to overtraining and sports injuries. A crucial difference was seen, especially after 8 weeks of training in the width of the aerobic window assessed by respectively the threshold and the angular method. The width of the AW was significantly larger across all horses for the angular method when compared to the threshold method. This emphasises the fact that the mathematical approach that is chosen to deduce training parameters becomes more and more important as the horse gains more condition and that the angular method respects more the individual shape of the lactate curves and thus takes into account subtle individual physiological differences. Since this study is the first to apply such an approach, no comparison can be made with results obtained in other animal species or human. More research is needed with that respect.

Some important remarks need to be kept in mind when further developing LMS test protocols: the possible protocol-dependency of the obtained LMS-test parameters; the pursuit of maximum reliability of the obtained LMS-test parameters to monitor training effects and the validation of the fact that the LMS is a good estimation of the MLSS.

For human athletes, several studies report protocol-dependency of the results of the LMS test (Carter et al., 1999; Ribeiro et al., 2009). However multiple recently conducted studies that investigated protocol dependency, show that it can be controlled (Smith et al., 2002).

Smith et al. (2002) showed that LMS determination was not dependent upon the hyperlactatemia-induction protocols, which precede the incremental step protocol. However, Zagatto et al. (2014) criticised Smith’s study and stated that there is an effect of the hyperlactatemia-protocol on obtained LMS parameters, in case very intense exercise steps are used to induce the hyperlactatemia (Zagatto et al., 2014). Nevertheless, they did not report the degree of induced hyperlactatemia (Messias et al., 2017). In our study, a clear hyperlactatemia-threshold was set: a blood lactate concentration >5 mmol/L. Therefore, horses weren’t challenged to maximal exercise prior to start-up of the incremental phase of the LMS protocol. Physiologically it can be questioned whether challenging “exhaustion” hyperlactatemia actually represents the situation that occurs during training and competition. Most likely this is not the case, therefore, choosing a cut-off value that is more physiological, is a better way. Another approach is to imply a certain recovery period after induction of pronounced hyperlactatemia, to prevent horses from needing to start-off with the incremental stage, right after completing exercise until exhaustion. Certainly, the key question then remains: “how long does such a recovery period need to be?”.

The recovery phase, which allows time for the lactate transposition from cells to the bloodstream, was very short in our study protocol (±1 min, allowing for drawing blood for lactate assessment). Many human study protocols involve a rather long recovery period (8 min or longer) in follow-up of the hyperlactatemia induced by exercise until exhaustion (Tegtbur et al., 1993). Recently it has been shown that this fixed period is not ideal and therefore application of individual recovery lengths, governed by serially measured blood lactate concentrations during recovery has been suggested (Messias et al., 2017). In horses, only the study of Gondim et al. (2007) used an individualised duration of the applied recovery period, the duration of which was based on the results of previously performed lactate time to peak tests (Gondim et al., 2007). Obviously, when exercise is performed until exhaustion, immediate start-up of an incremental protocol is not feasible. On another note, this recovery period does represent an additional variable in the LMS test, because body systems are allowed to adapt and recover and so, it means that the incremental phase of the LMS test is started-up at an interindividual different momentum in that recovery phase, in case a fixed and long duration recovery period is allowed. No doubt, this creates more variability in test results, because start-up of the incremental stages will occur for each individual, at a different stage of the recovery process. The latter is prevented by choosing a cut-off value of 5 mmol/L for the hyperlactatemia induction phase, which is an acceptable value for both poorly and well-trained horses (Courouce, 1999). Our study results show that by not inducing pronounced hyperlactatemia prior to start-up of the incremental protocol, the application of a real recovery phase is not needed. The *U*-shaped curve shows that horses further decline in their blood lactate concentration during the first incremental steps of the LMS protocol, despite the fact that they start-off from a hyperlactatemia. This is a more physiological approach that mimics real training and competition situations and it renders the application of a recovery period unnecessary. Governing duration of recovery periods by serial blood lactate measurements can be questioned as well, in view of the fact that the concept of recovery status is most probably illustrated by the interplay of many more physiological parameters than blood lactate and heart rate *per se*.

In the third and final stage of the LMS, which is the incremental step phase, a lot of protocol parameters can be changed, such as starting speed of the incremental steps, duration of the steps, etc., however, more research is needed. First, lower starting velocities can allow for a faster lactate clearance resulting in a lower LMS, leading to underestimation of the performance capacity of the horse (Carter et al., 1999). Ribeiro et al. (2003) showed no influence of stage length on LMS in trained swimmers (Ribeiro et al., 2003).

Not all existing research is equivocal on the capacity of differing LMS protocols to assess MLSS values and thus advances in performance capacity. A majority of human studies found a significant change of the LMS after training, and this for several different sports disciplines (Simões et al., 2009; Knoepfli-Lenzin, 2011; Sotero et al., 2011). Likewise, the current study shows clear training effects on multiple LMS test parameters in horses. However one single study reports no changes in LMS test parameters after weeks of endurance training, while the MLSS changed significantly, at least when an identical LMS-protocol was applied before and after training (Carter et al., 1999). These researchers suggest that after training, intensities of the incremental stages of the LMS must be modified (Carter et al., 1999). However, in our study, despite the fact that an identical LMS protocol was used before and after 8 weeks of training, a clear decrease was seen in LMS. Therefore, in future research, most probably the intense pre-phase to induce hyperlactatemia and the AW, needs further attention, rather than intensive adaptation of the post-training LMS test protocol. It can be expected that by increasing the intensities of the consecutive LMS steps, after training, the flattening of the *U*-shaped curve becomes less pronounced, allowing for a “sharper” delineation of the aerobic window, however, also influencing obtained performance parameters. Trying to assess how to adapt the post-training LMS protocol will not be easy. However large increases of power/speed may hinder precise determination of the MLSS, whereas too small incremental steps (with the same stage length) will predispose to the fact that the blood lactate concentration will drop causing a left-shift of the curve, especially in highly trained athletes.

In contrast to the SET-parameters, the LMS showed a good correlation (*r* = 0.93) with the V_MLSS_ before the start of the training period, however after 8 weeks of training the LMS had the tendency to underestimate the MLSS (illustrated in Figure 6, mean bias: −0.97 km/h) which is in accordance with human studies (Knoepfli-Lenzin, 2011). Maybe, this can be addressed by applying a certain conversion factor on the obtained post training LMS value. More research is needed with that respect. The correlation coefficient for LMS vs. V_MLSS_ (*r* = 0.65) was still high according to equine and mice studies after 8 weeks of training, indicating that the LMS is a promising tool to predict the V_MLSS_ with some adjustments to the protocol (Miranda et al., 2014; Rodrigues et al., 2016). Other studies showed a good correlation between the LMS and V_MLSS_ with correlation coefficients of 0.67–0.84 in mice and 0.7 in horses (Miranda et al., 2014; Rodrigues et al., 2016). Important to notice is that even when the MLSS would have been determined even more precisely, the SET-parameters would still overestimate the MLSS and horses would not be able to maintain this speed for 30 min without an increase of >1 mmol/L whole blood lactate concentration, leading to an increased risk for injury development. During the validation of the LMS at 100% LMS there was respectively no increase in blood lactate concentration more than 1mmol/L, while an increase of >1 mmol/L at 110% LMS was present in the majority of the mares, like initially shown by Tegtbur et al. (1993). Therefore the tested LMS protocol provides an accurate estimation of the V_MLSS_, like shown in several human studies (MacIntosh and Shane, 2002; Sotero et al., 2009; Knoepfli-Lenzin, 2011) and in a single study performed with horses (Soares et al., 2014). The V_MLSS_ was determined starting from the individual LMS-values of the horses. An additional session of 105% LMS could have provided even more accurate correlations.

Bland-Altman concordance analyses showed the best concordance between the V_MLSS_ and the LMS, followed by VLa_1_,_5_, VLa_2_, and VLa_4_. The mean bias and 95% agreement CI were considered acceptable for LMS in the field, since a mean difference of 1.04 km/h before training and −0.97 km/h after training is a quite precise estimation for field exercise testing. An acceptable mean bias of 1 km/h was chosen beforehand because riding horses more precisely with less than 1 km/h of variance is really difficult in the field. This difference has limited influence when comparing exercise capacity or external load prescription. One other equine study that validated the LMS-test showed a good concordance between the protocols with a mean bias of 0.5 km/h and a 95%CI of 1km/h; however this was a treadmill study (Miranda et al., 2014). Protocol adjustments are necessary for SET-thresholds in order to predict the V_MLSS_ because important mean biases were detected for SET-parameters. The VLa_4_ can be considered as an invalid method to predict the V_MLSS_. No fixed and proportional bias was detected for the LMS-test after 8 weeks of training compared to the MLSS. However, pre-training, a proportional and acceptable fixed bias (1.08 km/h) was seen (Restan and Cerqueira, 2019). Other equine and mice studies showed similar results and a wide variability of individual results in their plots (Miranda et al., 2014; Soares et al., 2014; Rodrigues et al., 2016).

Several human studies showed that even shorter versions of the LMS test are capable to estimate the MLSS, so maybe it is not necessary to complete the entire LMS-protocol to avoid the risk of occurrence of sports injuries (Simões et al., 2009; Sotero et al., 2011; Miyagi et al., 2013). More research is needed with that respect.

Regarding the set-up of training protocols: Training velocities around the LMS correlate with their individual lactate threshold; training velocities in the range of 10% below the AW_<LMS_ provide a complete aerobic training to that horse; and velocities in the range of 10% above the AW_>*LMS*_ challenge that specific horse anaerobically without creating overtraining. However, more research is needed with that respect and adaptations in function of the different equestrian disciplines would be interesting.

The results of the current study have also shown that during a LMS test, unlike during a classical incremental SET, horses do not need to perform maximal exercise. This means that the horses do not have to be pushed to extreme speeds, whereas in a SET, the horses have to keep running until maximal velocities. This is reflected in the significantly lower maximum blood lactate concentration achieved during the LMS when compared to the SET. Due to the fact that the horses have to exercise at maximum effort, they have a higher risk of sustaining injuries during the SET, ranging from musculoskeletal mishaps, to injuries at the level of the respiratory system, also rendering horse owners less prone to subject their horses to such SETs. Therefore, it seems that the classical SET is not optimal from an animal welfare point of view to assess aerobic capacity and training evolution. The LMS is much more eligible for that purpose. With a basic effort made by the horse, determination of the training level can be made under natural conditions and, in this way, a tailored training schedule can be set-up with a minimum risk for development of sports injuries.

Main study limitations: Protocol dependency of the performance parameters cannot be excluded. A limited number of horses was included in the study. The estrous cycle phases and its possible influence on the performance parameters were not determined. Some physiological determinants of performance, such as efficiency of movement and VO2_Max_ were not determined, because of insufficient reproducibility/validity that exists for these techniques for field testing in horses at this point (Betros et al., 2002; van Erck et al., 2007; Allen et al., 2016; Peyré-Tartaruga and Coertjens, 2018). This study provides an approximation of the V_MLSS_ and did not apply the MLSS golden standard method because this is not feasible in the field. No 105% LMS run was included in the current study. It would have been interesting to include such a step.

## Conclusion

This modified LMS test protocol is a valuable tool for assessing the aerobic window of individual horses at lower speed and blood lactate concentrations compared to SETs, which is an important factor from an animal welfare point of view. The LMS-test is easy to implement, valuable for field exercise testing and entails the challenge of the blood lactate-steady state in contrast to application of fixed thresholds. With that respect, the study clearly shows that VLa_4_ is an unreliable cut-off value to assess the aerobic capacity of a horse. The current study shows a better correlation between the V_MLSS_ and the LMS compared to the SET parameters, which up until now, is the most applied test protocol for monitoring exercise capacity in equine athletes. Bland-Altman analyses showed that the LMS-test provides a good estimation of the V_MLSS_ with an acceptable mean bias. Training clearly flattens the *U*-shaped LMS curve. The LMS is reached at an earlier stage and maintained for a longer time in trained horses, illustrated by the increased AW. The 40° angular method is the most solid mathematical LMS curve approach to determine the AW. The AW is a promising tool allowing for proper deduction of professional and effective training advice, tailored to the fitness level of that specific horse. The LMS protocol can be further adapted, especially post-training, however, the approach of inducing modest hyperlactatemia prior to start-off of the incremental LMS stages and omitting the inclusion of a per-test recovery period greatly contributes to its robustness.

## Data Availability Statement

The original contributions presented in the study are included in the article/supplementary material, further inquiries can be directed to the corresponding author/s.

## Ethics Statement

The animal study was reviewed and approved by the Animal Ethics Committee of the Ghent University (EC 2016/40).

## Author Contributions

LD contributed to data collection, analysis, and interpretation and writing and revision of the manuscript. CD and BB were involved in study setup, study design, data collection, analysis, and interpretation, and writing and revision of the manuscript. CM and CV contributed to data collection and preparation for analysis and reviewing of the manuscript. LP contributed to data collection and study design and reviewing of the manuscript. YG, FV, and DD contributed to data analysis and interpretation, the visual design of figures, and reviewing of the manuscript. JO and GH contributed to the study setup and its execution and reviewing of the manuscript. MO performed the Equinosis Q system analyses with Lameness Locator Software and reviewing of the manuscript. All authors read and approved the final manuscript.

## Conflict of Interest

The authors declare that the research was conducted in the absence of any commercial or financial relationships that could be construed as a potential conflict of interest.

## Publisher’s Note

All claims expressed in this article are solely those of the authors and do not necessarily represent those of their affiliated organizations, or those of the publisher, the editors and the reviewers. Any product that may be evaluated in this article, or claim that may be made by its manufacturer, is not guaranteed or endorsed by the publisher.

## References

[B1] Allen K. J. van Erck-Westergren E. Franklin S. H. (2016). Exercise testing in the equine athlete. *Equine Vet. Educ*. 28 89–98. 10.1111/eve.12410

[B2] Alves J. Santos A. Brites P. Ferreira-Dias G. (2012). Evaluation of physical fitness in police dogs using an incremental exercise test. *Comp. Exerc. Physiol*. 8 219–226. 10.3920/CEP12027 29510743

[B3] Arratibel-Imaz I. Calleja-González J. Emparanza J. I. Terrados N. Mjaanes J. M. Ostojic S. M. (2016). Lack of concordance amongst measurements of individual anaerobic threshold and maximal lactate steady state on a cycle ergometer. *Phys. Sportsmed*. 44 34–45. 10.1080/00913847.2016.1122501 26578151

[B4] Arratibel-Lmaz I. Calleja-González J. Terrados N. (2017). Validity of blood lactate measurements between the two LactatePro versions. *Arch. Med. Del. Deport*. 34 86–91.

[B5] Aunola S. Rusko H. (1992). Does anaerobic threshold correlate with maximal lactate steady-state? *J. Sports Sci*. 10 309–323. 10.1080/02640419208729931 1387688

[B6] Bacon L. (1999). Evaluating a test protocol for predicting maximum lactate steady state. *J. Sport Med. Phys. Fit*. 39 300–308. 10726430

[B7] Beneke R. (1995). Anaerobic threshold, individuale anaerobic threshold, and maximal lactate steady state in rowing. *Med. Sci. Sports Exerc*. 27 863–867. 7658947

[B8] Betros C. L. McKeever K. H. Kearns C. F. Malinowski K. (2002). Effects of ageing and training on maximal heart rate and VO2max. *Equine Vet. J. Suppl*. 34 100–105. 10.1111/j.2042-3306.2002.tb05399.x 12405667

[B9] Bourgela M. Blais D. M. M. (1991). Reproducibility and validity of VLA4 in Standardbred Pacer Horses on track. *Equine Exerc. Physiol*. 3 196–201.

[B10] Broeckx S. Y. Martens A. M. Bertone A. L. Van Brantegem L. Duchateau L. Van Hecke L. (2019). The use of equine chondrogenic-induced mesenchymal stem cells as a treatment for osteoarthritis: as randomised, double-blinded, placebo-controlled proof-of-concept study. *Equine Vet. J*. 51 787–794. 10.1111/evj.13089 30815897 PMC6850029

[B11] Brooks G. A. (2020). Lactate as a fulcrum of metabolism. *Redox Biol*. 35:101454. 10.1016/j.redox.2020.101454 32113910 PMC7284908

[B12] Campos E. Z. Nordsborg N. B. Da Silva A. S. R. Zagatto A. M. Neto J. G. Andrade V. L. (2014). The response of the lactate minimum test to a 12-week swimming training. *Motriz Rev. Educ. Fis*. 20 286–291. 10.1590/S1980-65742014000300007

[B13] Carter H. Jones A. M. Doust J. H. (1999). Effect of 6 weeks of endurance training on the lactate minimum speed. *J. Sports Sci*. 17 957–967. 10.1080/026404199365353 10622356

[B14] Clemente Suárez V. J. González-Ravé J. M. (2014). Four weeks of training with different aerobic workload distributions - Effect on aerobic performance. *Eur. J. Sport Sci*. 14(Suppl.1), 1–7. 10.1080/17461391.2011.635708 24444193

[B15] Courouce A. (1999). Field exercise testing for assessing fitness in French standardbred Trotters. *Vet. J*. 157 112–122. 10.1053/tvjl.1998.0302 10204407

[B16] Crotty N. M. Boland M. Mahony N. Donne B. Fleming N. (2021). Reliability and Validity of the Lactate Pro 2 Analyzer. *Meas. Phys. Educ. Exerc. Sci*. 25 202–211. 10.1080/1091367X.2020.1865966

[B17] Cunha R. R. Najara V. Cunha D. C. Moreira S. R. Kokubun E. Campbell C. S. (2009). Determination of the lactate threshold and maximal blood lactate steady state intensity in aged rats. *Cell Biochem. Funct*. 27 351–357. 10.1002/cbf 19585487

[B18] Davie A. L. Evans D. J. (2000). Blood Lactate Responses to Submaximal Field Exercise Tests in Thoroughbred Horses. *Vet. J*. 159 252–258. 10.1053/tvjl.1999.0420 10775469

[B19] De Mare L. Boshuizen B. Plancke L. De Meeus C. De Bruijn M. Delesalle C. (2017). Standardized exercise tests in horses: Current situation and future perspectives. *Vlaams Diergeneeskd Tijdschr*. 86 63–72. 10.21825/vdt.v86i2.16290

[B20] Donovan C. M. Brooks G. A. (1983). Endurance training affects lactate clearance, not lactate production. *Am. J. Physiol. Endocrinol. Metab*. 7 83–92. 10.1152/ajpendo.1983.244.1.e83 6401405

[B21] Dubreucq C. Chatard J. C. Courouce A. Auvinet B. (1995). Reproducibility of a standardised exercise test for Standardbred trotters under field conditions. *Equine Vet. J*. 27 108–112. 10.1111/j.2042-3306.1995.tb04900.x

[B22] Dyson S. (2000). Lameness and Poor Performance in the Sports Horse: Dressage, Show Jumping and Horse Trials (Eventing). *2000 AAEP Conv. Proc*. 46 308–315.

[B23] Evans D. L. Harris R. C. Snow D. H. (1993). Correlation of racing performance with blood lactate and heart rate after exercise in Thoroughbred horses. *Equine Vet. J*. 25 441–445. 10.1111/j.2042-3306.1993.tb02987.x 8223377

[B24] Ferasin L. Marcora S. (2009). Reliability of an incremental exercise test to evaluate acute blood lactate, heart rate and body temperature responses in Labrador retrievers. *J. Comp. Physiol. B Biochem. Syst. Environ. Physiol*. 179 839–845. 10.1007/s00360-009-0367-z 19455341

[B25] Foxdal P. Sjödin A. Sjödin B. (1996). Comparison of blood lactate concentrations obtained during incremental and constant intensity exercise. *Int. J. Sports Med*. 17 360–365. 10.1055/s-2007-972861 8858408

[B26] Gondim F. J. Zoppi C. C. Pereira-da-Silva L. de Macedo D. V. (2007). Determination of the anaerobic threshold and maximal lactate steady state speed in equines using the lactate minimum speed protocol. *Comp. Biochem. Physiol. A Mol. Integr. Physiol*. 146 375–380. 10.1016/j.cbpa.2006.11.002 17234441

[B27] Gramkow H. L. Evans D. L. (2006). Correlation of race earnings with velocity at maximal heart rate during a field exercise test in Thoroughbred racehorses. *Equine Vet. J*. 38(Suppl.36), 118–122. 10.1111/j.2042-3306.2006.tb05526.x 17402405

[B28] Hamlin M. J. Shearman J. P. Hopkins W. G. (2002). Changes in physiological parameters in overtrained Standardbred racehorses. *Equine Vet. J*. 34 383–388. 10.2746/042516402776249146 12117111

[B29] Hauser T. Adam J. Schulz H. (2014). Comparison of selected lactate threshold parameters with maximal lactate steady state in cycling. *Int. J. Sports Med*. 35 517–521. 10.1055/s-0033-1353176 24227122

[B30] Heck H. Mader A. Hess G. Mucke S. Muller R. Hoflmann W. (1985). Justification of the 4 mmol/L lactate threshold. *Int. J. Sport Med*. 6 117–130. 10.1055/s-2008-1025824 4030186

[B31] Impellizzeri F. M. Marcora S. M. Coutts A. J. (2019). Internal and external training load: 15 years on. *Int. J. Sports Physiol. Perform*. 14 270–273. 10.1123/ijspp.2018-0935 30614348

[B32] Jacobs I. Kaiser P. (1982). Lactate in blood, mixed skeletal muscle, and FT or ST fibres during cycle exercise in man. *Acta Physiol. Scand*. 114 461–466. 10.1111/j.1748-1716.1982.tb07010.x 7136776

[B33] Janeba M. Yaeger D. White R. Stavrianeas S. (2010). The dmax method does not produce a valid estimate of the lactate threshold. *J. Exerc. Physiol. Online* 13 50–57.

[B34] Knoepfli-Lenzin C. U. B. (2011). Lactate minimum is valid to estimate maximal lactate steady state in moderately and highly trained subjects. *Strength Cond*. 25 1355–1359. 10.1519/JSC.0b013e3181d6dbf4 21522075

[B35] Kohrt W. M. O’Connor J. S. S. J. S. (1987). Longitudinal assessment of responses by triathletes to swimming, cycling, and running. *Med. Sci. Sports Exerc*. 21 569–575. 2607947

[B36] Leleu C. Cotrel C. Courouce-Malblanc A. (2005). Relationships between physiological variables and race performance in French standardbred trotters. *Vet. Rec*. 156 339–342. 10.1136/vr.156.11.339 15789646

[B37] Lillo-Bevia J. R. Moran-Navarro R. Martinez-Cava A. Cerezuela V. Pallares J. G. A. (2018). 1-day maximal lactate steady-state assessment protocol for trained cyclists. *J. Sci. Cycl*. 7 9–16. 10.28985/180630.jsc.03

[B38] Lindner A. E. (2010b). Relationships between racing times of standardbreds and v4 and v200. *J Anim Sci*. 88 950–954. 10.2527/jas.2009-2241 19933440

[B39] Lindner A. E. (2010a). Maximal lactate steady state during exercise in blood of horses. *J. Anim. Sci*. 88 2038–2044. 10.2527/jas.2009-2693 20190168

[B40] Ludbrook J. (2002). Statistical techniques for comparing measurers and methods of measurement: a critical review. *Clin. Exp. Pharmacol. Physiol*. 29 527–536. 10.1046/j.1440-1681.2002.03686.x 12060093

[B41] MacIntosh B. R. Shane E. S. K. (2002). The Lactate Minimum Test for Cycling: estimation of the Maximal Lactate Steady State. *Can. J. Appl. Physiol*. 27 232–249. 10.1139/h02-014 12180316

[B42] Manzo A. Ootaki Y. Ootaki C. Kamohara K. Fukamachi K. (2009). Paper Comparative study of heart rate variability between healthy human subjects and healthy dogs, rabbits and calves. *Lab. Anim*. 43 41–45. 10.1258/la.2007.007085 19001066

[B43] Messias L. H. D. Gobatto C. A. Beck W. R. Manchado-Gobatto F. B. (2017). The lactate minimum test: Concept, methodological aspects and insights for future investigations in human and animal models. *Front. Physiol*. 8:389. 10.3389/fphys.2017.00389 28642717 PMC5463055

[B44] Miranda M. C. P. C. Queiroz-Neto A. Silva-Júnior J. R. Pereira M. C. (2014). Comparison of the lactate minimum speed and the maximal lactate steady state to determine aerobic capacity in purebred Arabian horses. *N Z Vet. J*. 62 15–20. 10.1080/00480169.2013.815103 23869425

[B45] Miyagi W. E. Leite J. V. M. Zagatto A. M. (2013). Influence of the selection from incremental stages on lactate minimum intensity; a pilot study. *Braz J. K. Hum. Perf*. 15 715–725.

[B46] Palmer A. S. Potteiger J. A. Nau K. L. Tong R. J. (1999). A 1-day maximal lactate steady-state assessment protocol for trained runners. *Med. Sci. Sports Exerc*. 31 1336–1341. 10.1097/00005768-199909000-00016 10487377

[B47] Peyré-Tartaruga L. A. Coertjens M. (2018). Locomotion as a powerful model to study integrative physiology: Efficiency, economy, and power relationship. *Front. Physiol*. 9 1–16. 10.3389/fphys.2018.01789 30618802 PMC6297284

[B48] Płoszczyca K. Jazic D. Piotrowicz Z. Chalimoniuk M. Langfort J. Czuba M. (2020). Comparison of maximal lactate steady state with anaerobic threshold determined by various methods based on graded exercise test with 3-minute stages in elite cyclists. *BMC Sports Sci. Med. Rehabil*. 12 1–9. 10.1186/s13102-020-00219-3 33292555 PMC7672951

[B49] Poole D. C. Erickson H. H. (2008). *Cardiovascular Function and Oxygen Transport: Responses to Exercise and Training*. First Edit. Amsterdam: Elsevier Ltd, 10.1016/B978-070202857-1.50012-3

[B50] Restan A. Z. Cerqueira J. A. (2019). Lactate and glucose thresholds and heart rate deflection points for Beagles during INTENSE exercise. *Am. J. Vet. Res*. 80:3. 10.2460/ajvr.80.3.284 30801212

[B51] Ribeiro L. Balikian P. Malachias P. B. V. (2003). Stage Length, spline function and lactate minimum swimming speed. *J. Sport Med.* P*hys. Fit*. 43 312–318. 14625512

[B52] Ribeiro L. F. P. Gonçalves C. G. S. Kater D. P. Lima M. C. S. Gobatto C. A. (2009). Influence of recovery manipulation after hyperlactemia induction on the lactate minimum intensity. *Eur. J. Appl. Physiol*. 105 159–165. 10.1007/s00421-008-0885-5 18853175

[B53] Rodrigues N. A. Torsoni A. S. Fante T. Reis I. G. M. (2016). Claudio Alexandre Manchado-Gobatto FB. Lactate minimum underestimates the maximal lactate steady-state in swimming mice. *Appl. Physiol. Nutr. Metab*. 42 1–25. 10.1139/apnm-2016-0198 28006434

[B54] Rogers B. Giles D. Draper N. Hoos O. Gronwald T. (2021). A New Detection Method Defining the Aerobic Threshold for Endurance Exercise and Training Prescription Based on Fractal Correlation Properties of Heart Rate Variability. *Front. Physiol*. 11:596567. 10.3389/fphys.2020.596567 33519504 PMC7845545

[B55] Santos L. Gonzalez V. Iscar M. Brime J. I. Fernandez-Rio J. Egocheaga J. (2010). A new individual and specific test to determine the aerobic–anaerobic transition zone (santos test) in competitive judokas. *J. Cond. Res*. 24 2419–2428. 10.1519/JSC.0b013e3181e34774 20802284

[B56] Serrano M. G. Evans D. L. Hodgson J. L. (2002). Heart rate and blood lactate responses during exercise in preparation for eventing competition. *Equine Vet. J. Suppl*. 34 135–139. 10.1111/j.2042-3306.2002.tb05406.x 12405674

[B57] Siahkouhian M. Azizan S. Roohi B. N. A. (2012). new approach for the determination of anaerobic threshold: Methodological survey on the modified Dmax method. *J. Hum. Sport Exerc*. 7 599–607. 10.4100/jhse.2012.72.23

[B58] Simões H. G. Hiyane W. C. Sotero R. C. Pardono E. Puga G. M. L. (2009). Polynomial modeling for the identification of lactate minimum velocity by different methods. *J. Sport Med. Phys. Fit*. 49 14–18. 19188890

[B59] Singer E. R. Barnes J. Saxby F. Murray J. K. (2008). Injuries in the event horse: Training versus competition. *Vet. J*. 175 76–81. 17204438 10.1016/j.tvjl.2006.11.009

[B60] Smith M. F. Balmer J. Coleman D. A. Bird S. R. Davison R. C. R. (2002). Method of lactate elevation does not affect the determination of the lactate minimum. *Med. Sci. Sports Exerc*. 34 1744–1749. 10.1097/00005768-200211000-00009 12439078

[B61] Soares O. A. B. Ferraz G. C. Martins C. B. Dias D. P. M. Lacerda-Neto J. C. Queiroz-Neto A. (2014). Comparison of maximal lactate steady state with V2, V4, individual anaerobic threshold and lactate minimum speed in horses. *Arq. Bras. Med. Vet. e Zootec*. 66 39–46. 10.1590/S0102-09352014000100007

[B62] Sotero R. C. Cunha V. N. C. Madrid B. Sales M. M. Moreira S. R. (2011). Lactate minimum identification in youth runners through a track test of three incremental stages. *Rev. Bras. Med. Esporte*. 17 119–122.

[B63] Sotero R. C. Pardono E. Landwehr R. Campbell C. S. G. Simoes H. G. (2009). Blood glucose minimum predicts maximal lactate steady state on running. *Int. J. Sports Med*. 30 643–646. 10.1055/s-0029-1220729 19569005

[B64] Svedahl K. MacIntosh B. R. (2003). Anaerobic threshold: The concept and methods of measurement. *Can. J. Appl. Physiol*. 28 299–323. 10.1139/h03-023 12825337

[B65] Swensen T. C. Harnish C. R. Beitman L. Keller B. A. (1999). Noninvasive estimation of the maximal lactate steady state in trained cyclists. *Med. Sci. Sports Exerc*. 31 742–746. 10.1097/00005768-199905000-00019 10331897

[B66] Tegtbur U. Busse M. W. Braumann K. M. (1993). Estimation of an individual equilibrium between lactate production and catabolism during exercise. *Med. Sci. Sports Exerc*. 25 620–627. 10.1249/00005768-199305000-00015 8492691

[B67] Urhausen A. Coen B. Weiler B. Kindermann W. (1993). Individual anaerobic threshold and maximum lactate steady state. *Int. J. Sports Med*. 14 134–139. 10.1055/s-2007-1021157 8509241

[B68] van Erck E. Votion D.-M. Serteyn D. Art T. (2007). Evaluation of oxygen consumption during field exercise tests in Standardbred trotters. *Equine Comp. Exerc. Physiol*. 4 43–49. 10.1017/s1478061507776466

[B69] von Haaren B. Haertel S. Stumpp J. Hey S. Ebner-Priemer U. (2015). Reduced emotional stress reactivity to a real-life academic examination stressor in students participating in a 20-week aerobic exercise training: a randomised controlled trial using Ambulatory Assessment. *Psychol. Sport Exerc*. 20 67–75. 10.1016/j.psychsport.2015.04.004

[B70] Wahl P. Zwingmann L. Manunzio C. Wolf J. Bloch W. (2018). Higher Accuracy of the Lactate Minimum Test Compared to Established Threshold Concepts to Determine Maximal Lactate Steady State in Running. *Int. J. Sports Med*. 39 541–548. 10.1055/s-0044-102131 29775989

[B71] Wyon M. Redding E. (2003). Strengths and Weaknesses of Current Methods for Evaluating the Aerobic Power of Dancers. *J. Danc. Med. Sci*. 7 10–16.

[B72] Zagatto A. M. Padulo J. Müller P. T. G. Miyagi W. E. Malta E. S. Papoti M. (2014). Hyperlactemia induction modes affect the lactate minimum power and physiological responses in cycling. *J. Strength Cond. Res*. 28 2927–2934. 10.1519/JSC.0000000000000490 24736777

[B73] Zhou S. Weston S. B. (1997). Reliability of using the D-max method to define physiological responses to incremental exercise testing. *Physiol. Meas*. 18 145–154. 10.1088/0967-3334/18/2/005 9183808

[B74] Zwingmann L. Strütt S. Martin A. Volmary P. Bloch W. Wahl P. (2019). Modifications of the Dmax method in comparison to the maximal lactate steady state in young male athletes. *Phys. Sportsmed*. 47 174–181. 10.1080/00913847.2018.1546103 30408426

